# IL-1 Pathway Inhibition in Recurrent Pericarditis Management: Real-World Adoption of Corticosteroid Sparing in RESONANCE

**DOI:** 10.1016/j.jacadv.2025.102050

**Published:** 2025-08-15

**Authors:** Paul C. Cremer, Sushil A. Luis, Michael S. Garshick, Ajit Raisinghani, Brittany Weber, Dona Winnowski, JoAnn Clair, Vidhya Parameswaran, Allison Curtis, Allan L. Klein, John F. Paolini, RESONANCE Study Group

**Affiliations:** a Division of Cardiology, Bluhm Cardiovascular Institute, Departments of Medicine and Radiology, Northwestern University Feinberg School of Medicine, Chicago, Illinois, USA; b Department of Cardiovascular Medicine, Mayo Clinic, Rochester, Minnesota, USA; c Cardio-Rheumatology Program, Center for the Prevention of Cardiovascular Disease, NYU Langone Health, New York, New York, USA; d Leon H. Charney Division of Cardiology, Department of Medicine New York University School of Medicine, New York, New York, USA; e Division of Cardiology, Department of Medicine, Sulpizio Cardiovascular Center, University of California San Diego, San Diego, California, USA; f Division of Cardiovascular Medicine, Department of Medicine, Brigham and Women’s Hospital, Harvard Medical School, Boston, Massachusetts, USA; g Patient author, Pericarditis Alliance, Salem, Oregon, USA; h Department of Medical Affairs, Kiniksa Pharmaceuticals, Lexington, Massachusetts, USA; i Center for the Diagnosis and Treatment of Pericardial Diseases, Section of Cardiovascular Imaging, Department of Cardiovascular Medicine, Heart, Vascular and Thoracic Institute,, Cleveland, Ohio, USA

**Keywords:** autoinflammatory disease, interleukin-1, interleukin-1 inhibition, recurrent pericarditis, rilonacept

## Abstract

**Background:**

Recurrent pericarditis (RP) guidelines recommend second-line corticosteroids after aspirin/nonsteroidal anti-inflammatory drugs/colchicine, but complications of protracted use underscore the importance of corticosteroid-sparing strategies.

**Objectives:**

Given clinical trial evidence supporting interleukin (IL)-1 pathway inhibition and US Food and Drug Administration approval of rilonacept in RP, the objective was to assess temporal trends in corticosteroid-sparing treatment of RP in a multicenter registry.

**Methods:**

RESONANCE (REgiStry Of the NAtural history of recurreNt periCarditis in pEdiatric and adult patients; NCT04687358) combines retrospective and prospective data from clinical practice into a single observation period. Patients receiving treatment for idiopathic and postprocedural RP were included. Demographics and treatment intensifications were quantified.

**Results:**

Of 313 patients with aspirin/nonsteroidal anti-inflammatory drugs/colchicine treatment (median disease duration 1.9 years; 1 preenrollment recurrence), 44% (n = 138) intensified treatment. Before rilonacept approval in RP, 71% (n = 10) of patients intensified to corticosteroids for second-line therapy, and 29% (n = 4) intensified to IL-1 pathway inhibition. After rilonacept approval, the proportion of patients intensifying to second-line IL-1 pathway inhibition increased to 64% by 2023 (n = 27; rilonacept: 57%, anakinra: 7%), whereas the proportion of patients intensifying to corticosteroids decreased to 33% (n = 14), *P* = 0.02. Approximately 59% (n = 35) of second-line corticosteroid initiators later transitioned to IL-1 pathway inhibition. In patients starting second-line IL-1 pathway inhibition, 5% (n = 3, rilonacept) and 25% (n = 4, anakinra) subsequently used corticosteroids for >30 days.

**Conclusions:**

In RESONANCE, IL-1 pathway inhibition increasingly replaced corticosteroids as second-line therapy. Most patients starting corticosteroids transitioned to IL-1 pathway inhibition; few transitioned from second-line IL-1 pathway inhibition to long-term corticosteroids. These findings may inform treatment algorithms and patient-provider decision-making.

Approximately 40,000 patients in the United States have recurrent pericarditis (RP), a chronic autoinflammatory disease mediated by interleukin (IL)-1; of these, approximately 14,000 experience multiple recurrences.^1^, ^2^, ^3^, ^4^, ^5^ RP involves episodic flares characterized by debilitating chest pain, and patients experience substantial morbidity and reduced health-related quality of life due to the multiple flares and fear of future recurrences.^6^^,^^7^

RP is a persistent and burdensome disease which often warrants several years of treatment.^6^^,^^7^ For patients with incomplete response to first-line therapy (ie, high-dose [500-1000 mg every 6-8 hours] aspirin, nonsteroidal anti-inflammatory drugs [NSAIDs], and/or colchicine), the 2015 European Society of Cardiology (ESC) guidelines recommended corticosteroids as second-line therapy. Advanced targeted therapy (ie, IL-1 pathway inhibition) was restricted to third-line therapy (only after patients had failed or had not tolerated corticosteroids), based on the limited evidence available at the time.^8^ Corticosteroid use over the duration of the disease, however, entails serial tapering regimens which may increase the frequency of future recurrences and predispose patients to osteoporosis, worsening of diabetes, hypertension, and health-related quality of life consequences.^9^, ^10^, ^11^, ^12^ These complications underscore the need for an updated treatment algorithm utilizing corticosteroid-sparing strategies.

Clinical evidence reported after these guidelines had been published showed that IL-1α and IL-1β mediate the pathophysiology of RP through the inappropriate induction and activation, respectively, of the innate immune system in a self-perpetuating cycle of autoinflammation.^13^ The phase 3 trial RHAPSODY (Rilonacept Inhibition of Interleukin-1 Alpha and Beta for Recurrent Pericarditis: a Pivotal Symptomatology and Outcomes Study) demonstrated that rilonacept (IL-1α and IL-1β cytokine trap) effectively treated RP and, as monotherapy, reduced risk of recurrence in both third-line (after corticosteroids) and second-line (instead of corticosteroids) use, which led to the US Food and Drug Administration's (FDA's) expansion of therapeutic indications for rilonacept in March 2021 to include treatment of RP and reduction in risk of recurrence regardless of prior treatment.^14^^,^^15^ Subsequently, increased emphasis has been placed on IL-1 pathway inhibition as a potential second-line corticosteroid-sparing therapy, consistent with this clinical trial evidence.^1^^,^^16^, ^17^, ^18^, ^19^, ^20^

The RESONANCE (REgiStry Of the NAtural history of recurreNt periCarditis in pEdiatric and adult patients) is the first multicenter, U.S.-based, noninterventional, observational registry for patients with RP, designed to quantify historical trends in disease burden, treatment patterns, and outcomes. RESONANCE captures longitudinal clinical management data at 29 sites and is designed to enroll ∼500 patients for up to a 5-year prospective observation period (6 years total observation). This first report of data from RESONANCE examines treatment patterns at participating U.S.-based pericardial disease–dedicated programs, specifically, adoption of IL-1 pathway inhibition in a corticosteroid-sparing strategy from March 1, 2020, to December 31, 2023.

## Methods

### Study design

RESONANCE (NCT04687358) was launched in March 2021. RESONANCE includes sites with pericardial disease–dedicated programs and experience in treating RP in the United States. Sites who had participated in RHAPSODY and sites expressing interest in the registry were included; a decentralized site was provided to include patients who wished to participate in the registry remotely (Supplemental Figure 1).

Patients with a diagnosis of RP were enrolled into either active (recurrence ≤3 years of enrollment) or inactive (no recurrences ≤3 years of enrollment) cohorts. Inclusion criteria for the active cohort were a physician-confirmed diagnosis of RP (an incident acute pericarditis episode and ≥1 recurrence following the incident episode), a recurrence ≤3 years before inclusion, were under the care of a physician for RP, and were currently prescribed any medication to treat pericarditis (eg, aspirin, colchicine, NSAIDs, corticosteroids [prednisone and methylprednisolone], conventional disease-modifying antirheumatic drugs [csDMARDs: sulfasalazine, hydroxychloroquine, methotrexate, azathioprine, etc], or IL-1 pathway inhibition [anakinra or rilonacept]). Notable exclusion criteria were diagnosis of pericarditis secondary to tuberculosis, cancer (if not in full remission), post thoracic blunt trauma, myocarditis, systemic autoimmune diseases, and/or HIV. Informed consent (and assent from patients under legal consenting age) was obtained on site or via e-consent remotely from each eligible patient and/or their legally authorized representative/caregiver. Sites were encouraged to assess consecutive patients for inclusion; patients were approached sequentially by their provider at clinic visits and informed of RESONANCE to enable the observation of RP management of all patient types in proportion to their presentation. Data within this interval analysis focus solely on the active cohort; an “inactive” cohort is described in the Supplemental Methods.

**Figure 1:**
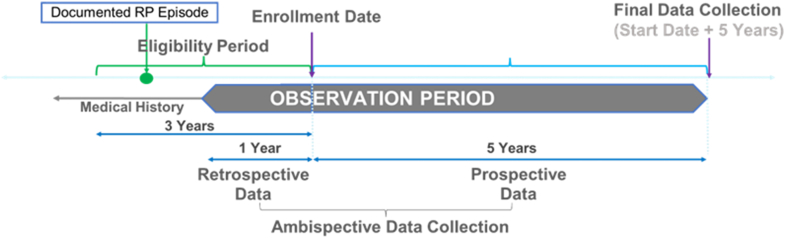
**Active Cohort Study Design** RESONANCE employs a hybrid data collection approach: up to 1-year retrospective data (the year prior to enrollment) are combined with prospective data into a single ambispective observation period. RP = recurrent pericarditis; RESONANCE = REgiStry Of the NAtural history of recurreNt periCarditis in pEdiatric and adult patients.

Deidentified data (anonymous to nonsite staff) from enrolled patients’ medical charts were extracted by site staff and entered into electronic case report forms. Retrospectively collected data from the incident acute pericarditis episode or up to 1 year before RESONANCE enrollment date were combined with prospectively collected data into a single ambispective observation period (Figure 1). Participants had prospective observational check-ins every 6 months (Supplemental Table 1).

Retrospective data collection included general disease data (eg, data on RP diagnosis, number of episodes in the observation period, other relevant conditions and comorbidities, 12-month prior surgical history, and RP medications) from the retrospective observation period (the period of 12 months before registry enrollment) and medical history data (eg, data on initial RP diagnosis, initial acute episode, and medications prescribed for first recurrence) from before the prospective observation period.

Baseline data (eg, patient characteristics/sociodemographic information, and RP–related medical history/treatment/medication data) were collected at enrollment.

Prospective data collection included data collected every 6 months, at standard of care appointments or 6-month chart reviews, regarding RP episode evolution, laboratory/diagnostic measures, and medication/medical history related to RP. Adverse events/safety data were not collected in the RESONANCE data set; however, reasons for treatment transitions were documented to provide additional context for any observed treatment patterns and to discern if any observed transitions were prompted by tolerability issues. Sample visual patient narratives are illustrated in Supplemental Figure 2.

For inactive patients, the observation period was purely retrospective, occurring between 3 and 5 years before enrollment in RESONANCE and captured data pertaining to only their final RP episode (Supplemental Figure 3).

RESONANCE was conducted in accordance with International Conference on Harmonization Good Clinical Practice, all applicable patient privacy requirements, and the ethical principles that are outlined in the Declaration of Helsinki 2013, including, but not limited to, central Institutional Review Boards (IRB), local IRB review and approval of the study protocol and any subsequent amendments, and investigator reporting requirements.

### Interval data analysis

The primary objectives of this interval analysis (observation period from March 1, 2020, to December 31, 2023) were to describe and characterize treatment patterns over time (ie, before vs after the availability of rilonacept in RP [April 2021 milestone]) in the subset of active cohort patients who received treatment with aspirin/NSAIDs/colchicine therapy (including those who intensified treatment) during that observation period and to identify trends in demographics, clinical characteristics, medication management choices, including trends in second-line and third-line treatment choices.

**Figure 2:**
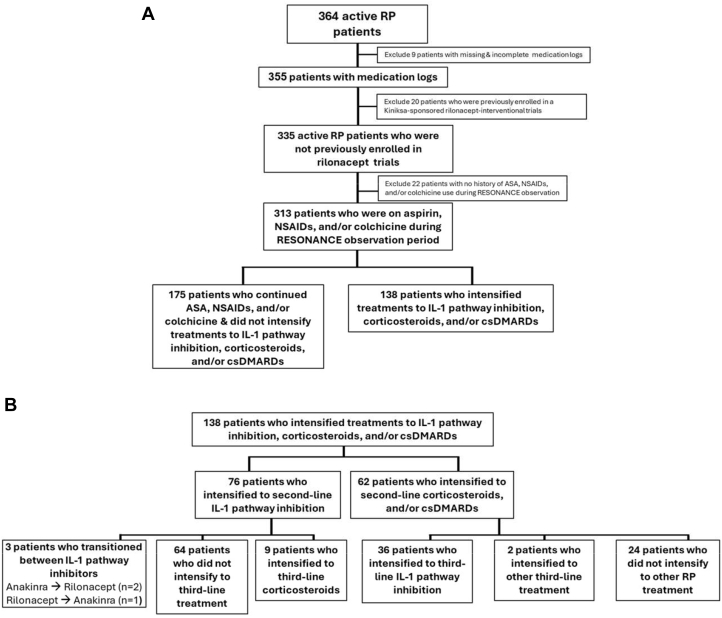
**Interval Analysis Patient Flow Chart** (A) Overall population for interval analysis (B) Subset of patients who intensified treatment from aspirin/NSAIDs/colchicine therapy. Across the entire interval analysis observation period, IL-1 pathway inhibition was the most commonly added pharmacotherapy for RP. ASA = aspirin; csDMARD = conventional disease-modifying antirheumatic drug; IL-1 = Interleukin-1; NSAID = nonsteroidal anti-inflammatory drug; tx = treatment; other abbreviations as in Figure 1.

All enrolled active cohort patients were included for demographics analysis. Failure of aspirin/NSAIDs/colchicine therapy was defined as intensification of treatment (add-on/switch) to corticosteroids, csDMARDs, or IL-1 pathway inhibition. Patients who intensified treatment from first-line aspirin/NSAIDs/colchicine to second-line therapy and contributed medication use data toward the interval analysis were included in the second-line treatment choice analysis. Patients who had either prior enrollment in RHAPSODY or missing medication logs, those who transitioned from aspirin/NSAIDs/colchicine to a short-term (<30 days) RP treatment followed by no other RP treatment, or those who did not intensify treatment beyond aspirin/NSAIDs/colchicine were excluded from the second-line treatment choice analysis (Figure 2A). Patients previously enrolled in rilonacept-interventional trials (including RHAPSODY) were excluded, as management within a clinical trial may not be indicative of real-world practice patterns.

Data were censored at the end of the interval analysis observation period, last check-in visit, or end of study; for patients who chose to exit RESONANCE before the end of the interval analysis observation period, all data were included in this analysis for their period of participation, and data were censored at their end of study visit.

For patients who intensified treatment beyond aspirin/NSAIDs/colchicine during the interval analysis observation period, intensification to second-line treatment was evaluated using the proportion of patients who added/switched to corticosteroids, csDMARDs, or IL-1 inhibitors during the observation period. The IL-1 pathway inhibitor use analysis included patients who switched to IL-1 pathway inhibition directly after aspirin/NSAIDs/colchicine (second line) or after intercurrent treatment with corticosteroids or csDMARDs (third line).

Descriptive statistics were used to describe pharmacologic treatment patterns. Normally distributed data are presented as mean ± SD; all other data are presented as median (Q1, Q3) and n (%). Categorical variables were compared using the chi-square test for independence; Fisher exact test was conducted to examine the association between treatment intensification patterns and comparative time periods. All analyses were performed using SAS Enterprise Guide 9.4 (SAS Institute, Inc), and a 2-tailed *P* < 0.05 was considered statistically significant.

## Results

### Patient/disease characteristics and treatment sequencing

A total of 409 patients (active cohort, n = 364 across 26 sites [8 of which were previous RHAPSODY sites]; median [Q1, Q3] time to last observation: 2.0 [1.0, 2.6] years) from 29 participating sites were enrolled in RESONANCE as of the end of 2023 (Supplemental Figure 1); 9 patients dropped out (median [Q1, Q3] follow-up time of 2.4 [2.0, 2.9] years). Per-site enrollment ranged from 1 to 66 patients, based on site volume and local practice. Select baseline patient and disease characteristics are reported in Table 1. Patients in the active cohort (n = 364) had a mean (SD) age of 45.6 (16.4) years at their index acute episode, were mostly women (60%, [n = 218/364]), most frequently had idiopathic/viral etiology (66%, [n = 239/364]), had a median (Q1, Q3) of 1 (1, 2) prior recurrence(s) at enrollment, a median (Q1, Q3) annualized recurrence rate of 2.0 (1, 3) at enrollment, and had a median (Q1, Q3) disease duration of 3.0 (1.8, 5.7) years at the end of the interval analysis observation period.

**Table 1:** Select Patient Demographic and Disease Characteristics

	All Patients (N = 364)	Active Patients With No Prior Enrollment in Rilonacept-Interventional Trials (n = 335)	Total Patients Who Were on Aspirin/NSAIDs/Colchicine (n = 313)	Patients Who Intensified From Aspirin/NSAIDs/Colchicine to Second-Line + Treatment (n = 138)	Patients Who Continued Aspirin/NSAIDs/Colchicine (n = 175)	*P* Value
Age^a^, y; mean ± SD	45.6 ± 16.4	45.5 ± 16.6	45.1 ± 16.4	46.0 ± 15.9	44.5 ± 16.9	0.44
Female, n (%)	218 (59.9)	200 (59.7)	188 (60.1)	82 (59.4)	106 (60.6)	0.84
White, n (%)	301 (82.7)	275 (82.1)	258 (82.4)	117 (84.8)	141 (80.6)	0.33
Etiology, n (%)						0.63
Idiopathic/viral pericarditis	239 (65.7)	224 (66.9)	208 (66.5)	98 (71.0)	110 (62.9)	
Postcardiac injury/postprocedural	43 (11.8)	40 (11.9)	39 (12.5)	17 (12.3)	22 (12.6)	
Other causes	17 (4.7)^c^	16 (4.8)	15 (4.8)	5 (3.6)	10 (5.7)	
Not reported	65 (17.9)	55 (16.4)	51 (16.3)	18 (13.0)	33 (18.9)	
Pericarditis disease duration at RP diagnosis^a^, y; median (Q1, Q3)	0.3 [0.1, 0.8]	0.3 [0.1, 0.8]	0.3 [0.1, 0.8]	0.2 [0.1, 0.4]	0.4 [0.1, 1.1]	0.72
Pericarditis disease duration at RESONANCE enrollment^a^^,^^b^, y; median (Q1, Q3)	1.9 [0.9, 4.3]	1.8 [0.8, 3.9]	1.7 [0.8, 3.8]	1.3 [0.6, 3.1]	2.0 [1.0, 4.6]	0.05
Pericarditis disease duration at the end of RESONANCE observation period^a^, y; median (Q1, Q3)	3.0 [1.8, 5.7]	2.8 [1.8, 5.1]	2.7 [1.8, 5.0]	2.6 [1.6, 4.4]	3.0 [1.9, 5.8]	0.10
Observation period, y, median (Q1, Q3); sum	2.0 [1.0, 2.6]; 702.6	1.9 [1.0, 2.6]; 635.9	1.9 [1.0, 2.6]; 595.5	2.0 [1.1, 2.7]; 269.5	1.9 [1.0, 2.6]; 326.0	0.39

NSAIDs = nonsteroidal anti-inflammatory drugs; Q1 = first quartile; Q3 = third quartile; RP = recurrent pericarditis; RESONANCE = REgiStry Of the NAtural history of recurreNt periCarditis in pEdiatric and adult patients.

a At the index acute episode; disease duration calculated as time since index acute episode.

b End of RESONANCE observation period for this interval analysis defined as end of study, last check-in visit, or December 31, 2023.

c Other causes include postoperative pericarditis (n = 2), stress/anxiety (n = 2), bacterial (non-TB) (n = 1), COVID vaccine-induced (n = 1), bronchitis/pneumonia (n = 1), post-pneumonia vaccine (n = 1), radiation (n = 1), bowel surgery (n = 1), vaccine-related (n = 1), acute fibrinous pericarditis (n = 1), cardiotoxic chemotherapy (n = 1), postatrial septal defect closure (n = 1), post-COVID-19 booster (n = 1), post-COVID vaccine (n = 1), and thyroid surgery (n = 1).

Of these 364 active cohort patients, 98% (n = 355/364) had nonmissing medication records; 5% (n = 20/364) were excluded due to prior participation in rilonacept-interventional trials (including RHAPSODY), 6% (n = 22/364) were excluded due to no medication history of aspirin/NSAIDs/colchicine, and 48% (n = 175/313) were excluded due to lack of treatment intensification beyond aspirin/NSAIDs/colchicine, resulting in 138 patients (median [Q1, Q3] time to last observation: 2.0 [1.1, 2.7] years) included in the second-line treatment choice analysis. Although patients who continued treatment with aspirin/NSAIDs/colchicine (n = 175; median [Q1, Q3] time to last observation: 1.9 [1.0, 2.6] years) had a longer pericarditis disease duration at RESONANCE enrollment than those who intensified treatment to second-line therapy during the interval analysis observation period (2.0 vs 1.3 years; *P* = 0.05), all other demographics and disease characteristics were similar (*P* > 0.05) between these 2 cohorts (Table 1).

### Second-line therapy before and after rilonacept approval in RP

Integrating across the entire interval analysis observation period, in the second-line intensification setting, IL-1 pathway inhibition was the most commonly added pharmacotherapy for RP (55% [n = 76/138]: subdivided as rilonacept 43% [n = 60/138], anakinra 12% [n = 16/138]), followed by corticosteroids (43% [n = 59/138]), then csDMARDs (2% [n = 3/138]) (Figure 2B). For rilonacept, the median [Q1, Q3] duration of treatment was 1.7 (1, 2.3) years; all patients initiated treatment according to label (loading and maintenance dosing); 80% of the patients on rilonacept were continuing on therapy at the time of data cutoff.

**Figure 3:**
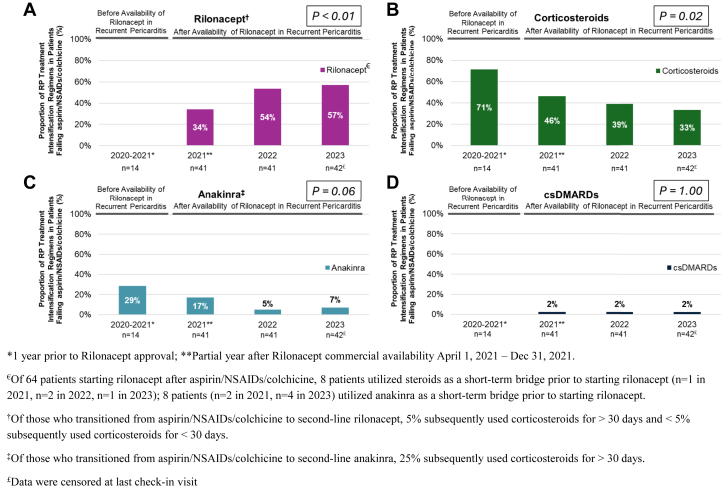
**Second-Line Treatment Choice Over Time in Patients Failing Aspirin/NSAIDs/Colchicine** Intensification to second-line treatment was evaluated using the proportion of patients who added/switched from aspirin/NSAIDs/colchicine to (A) rilonacept, (B) corticosteroids, (C) anakinra, and (D) csDMARDS during the observation period. Categorical variables were compared using the chi-square test for independence; Fisher exact test was conducted to examine the association between treatment intensification patterns and comparative time periods. Before the approval of rilonacept in RP most patients transitioned to second-line corticosteroids; after rilonacept approval for RP, the proportion of patients intensifying treatment to IL-1 pathway inhibition incrementally increased year-on-year to 64% in 2023. Abbreviations as in Figures 1 and 2.

Prior to the approval of rilonacept in RP (March 2021; commercial availability in April 2021), of patients intensifying treatment from aspirin/NSAIDs/colchicine to second-line therapy, 71% (n = 10/14) transitioned to corticosteroids, 29% (n = 4/14) transitioned to IL-1 pathway inhibition, and no patients transitioned to csDMARDs (Figure 3). During the time period after rilonacept approval for RP, of patients intensifying treatment from aspirin/NSAIDs/colchicine to second-line therapy, the proportion of patients intensifying treatment to IL-1 pathway inhibition incrementally increased year-on-year to 64% in 2023 (rilonacept: 57% [*P* < 0.01]; anakinra: 7% [*P* = 0.06]), whereas the proportion of patients intensifying treatment to corticosteroids significantly decreased over the same period to 33% (*P* = 0.02) in 2023 (Central Illustration); the proportion of patients intensifying treatment to csDMARDs remained constant at ∼2% (*P* = 1.00).

**Central Illustration:**
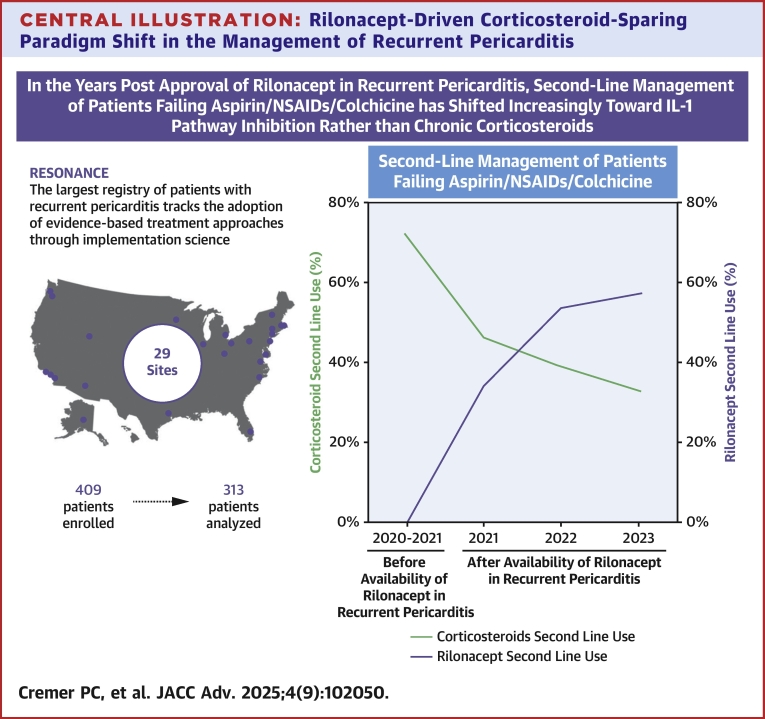
Rilonacept-Driven Corticosteroid-Sparing Paradigm Shift in the Management of Recurrent Pericarditis

### Durability of second-line treatment choice

Among those who received second-line IL-1 pathway inhibition, 88% (n = 67/76) did not change treatment to a different class of medication for third-line therapy, and 12% (n = 9/76) transitioned to third-line corticosteroids. Of those who transitioned from aspirin/NSAIDs/colchicine to second-line rilonacept, 5% (n = 3/60) subsequently used corticosteroids for >30 days during the remainder of the observation period, and 3% (n = 2/60) of patients subsequently used corticosteroids briefly for <30 days (for the management of a friction rub episode and pericardial effusion episode, respectively). Only 2% (n = 1/60) of patients who had received second-line rilonacept subsequently used third-line anakinra for >30 days. Of those who transitioned from aspirin/NSAIDs/colchicine to anakinra, 25% (n = 4/16) patients subsequently used corticosteroids for >30 days. Of patients who started anakinra as second-line therapy, 13% (n = 2/16) transitioned to rilonacept as third-line therapy.

Among those who advanced to second-line treatment with corticosteroids, 59% (n = 35/59) subsequently transitioned to IL-1 pathway inhibition as third-line therapy (54% [n = 32/59] to rilonacept [median time to IL-1 pathway initiation after initiation of corticosteroids was 4.2 months] and 5% [n = 3/59] to anakinra [median time to IL-1 pathway initiation after initiation of corticosteroids was 4.5 months]; the most common reasons for this transition from corticosteroids to IL-1 pathway inhibition were chest pain [71%; n = 25/35], more intense pain while breathing [14%; n = 5/35], and shortness of breath when reclining [11%; n = 4/35]). Approximately 3% (n = 1/59) of patients transitioned from second-line corticosteroids to a class of medications other than IL-1 pathway inhibitors (ie, csDMARDs) as third-line therapy. About one-third (39% [n = 23/59]) of all patients who received second-line corticosteroids did not intensify treatment to a subsequent third-line therapy.

Patients who initiated IL-1 pathway inhibition as second-line treatment (n = 76; median [Q1, Q3] time to last observation: 2.0 [1.1, 2.8] years) vs as third-line treatment (n = 36; median [Q1, Q3] time to last observation: 2.2 [1.5, 2.9] years) had mostly similar (*P* > 0.05) disease characteristics (Supplemental Table 2).

## Discussion

RP is a chronic disease that often requires long-term treatment. Although the 2015 ESC guidelines for the treatment of RP recommended broad immunosuppression with corticosteroid-based regimens as second-line therapy for patients failing aspirin/NSAIDs/colchicine, this recommendation, which was the state of the art at the time, was made prior to the evolution in knowledge about the efficacy of IL-1 pathway inhibition for the treatment of RP and the reduction in risk of recurrence.^8^^,^^14^^,^^15^^,^^20^, ^21^, ^22^ The increased risk of recurrence and complications associated with long-term corticosteroid use over the long duration of RP natural history (3.3 year median) underscore the importance of corticosteroid-sparing strategies.^23^^,^^24^ The adverse effects of corticosteroids are dose- and time-dependent; prolonged therapy with corticosteroids predisposes patients to an array of complications and iatrogenic consequences.^25^, ^26^, ^27^ Providers who utilize treatment with long-term corticosteroids have often opted for an episodic approach, with a focus on flare management with a brief course of suppressive corticosteroid treatment followed by a slow taper to minimize cumulative adverse effects; however, premature (while the underlying autoinflammation is still present) cessation of immunosuppression often results in recurrences due to insufficient inhibition of the underlying pathophysiology for the full disease duration.^4^^,^^5^^,^^28^, ^29^, ^30^ Corticosteroid-based treatment strategies, because they entail serial tapers to minimize cumulative adverse effects, are therefore associated with a high rate of recurrence and can worsen patient quality of life due to the recurrences themselves and the complexity and uncertainty of their disease course.^12^^,^^23^^,^^30^, ^31^, ^32^

To address the burden of corticosteroid-based treatment strategies, there was an expansion of clinical trial investigation of IL-1 pathway inhibitors in RP in the period after the 2015 ESC Guidelines.^15^^,^^33^, ^34^, ^35^ The AIRTRIP (Anakinra Treatment of Recurrent Idiopathic Pericarditis) study was the first clinical trial of IL-1 pathway inhibition in RP; it demonstrated that anakinra (with/without concomitant colchicine), when used third line in flaring colchicine-resistant corticosteroid-dependent idiopathic RP patients, significantly reduced the risk of recurrence compared with placebo; 90% (9/10) of patients receiving placebo experienced a recurrence vs 18% (2/11) in those receiving anakinra. Importantly, although the active arm was efficacious, approximately half (45%) of the patients receiving anakinra were also still receiving concomitant colchicine during the randomized-withdrawal period, as monotherapy was not obligatory in this seminal study.^33^ The design of AIRTRIP was consistent with the positioning of IL-1 pathway inhibition as third-line therapy in the 2015 ESC guidelines.^8^^,^^33^

The rilonacept clinical development program tested rilonacept not only as a third-line treatment (in corticosteroid-dependent patients) but also as a second-line treatment (in colchicine-resistant patients, instead of corticosteroids). In addition, RHAPSODY tested a broader patient population and required rilonacept to be given as obligatory monotherapy. Accordingly, while half of all patients in the pivotal phase 3 trial RHAPSODY had transitioned to rilonacept from corticosteroids in the traditional third-line approach, the other half had transitioned from NSAIDs/colchicine in a novel second-line approach. Efficacy of rilonacept and transition to rilonacept monotherapy were similar in both groups. RHAPSODY demonstrated the two-fold potential of rilonacept in corticosteroid sparing: as third-line therapy, to reduce the risks associated with prolonged exposure to corticosteroids for those already on them, and as second-line therapy, to obviate initiation of corticosteroid-based regimens while exhibiting a tolerable safety profile. The most common adverse events with rilonacept were injection-site reactions and upper respiratory tract infections.^15^ These data laid the foundations for the evolution in clinical management demonstrated in these real-world registry data.

Observational registry data have further elucidated implementation of IL-1 pathway inhibition in clinical practice. First, the IRAP (International, all-comers, multicenter Registry of Anakinra for Pericarditis), an international registry that captured observational data from 2014 to 2018, further affirmed in a real-world setting the efficacy of anakinra in colchicine-resistant/corticosteroid-dependent refractory pericarditis patients and demonstrated that the addition of once-daily anakinra (to ongoing NSAIDs/colchicine ± corticosteroid therapy) reduced rates of recurrences, emergency department admissions, hospitalizations, and corticosteroid dependence.^34^ This interval analysis of RESONANCE (which captured data onward from 2020 regardless of prior medication history) demonstrated that prior to the approval of rilonacept by the FDA in RP in March 2021 the majority (71%) of patients failing (ie, intensifying therapy beyond) aspirin/NSAIDs/colchicine received second-line therapy with corticosteroids; although anakinra and rilonacept were both already FDA-approved for other indications and therefore available clinically, only 29% were treated with IL-1 pathway inhibition, specifically, anakinra.^14^^,^^36^ Subsequently, after FDA approval of rilonacept for RP, for patients failing aspirin/NSAIDs/colchicine, clinicians at centers with pericardial disease–focused programs initiated IL-1 pathway inhibition instead of corticosteroids as a second-line therapy increasingly year-over-year during the observation period. This increase in second-line use of IL-1 pathway inhibition use was driven predominantly by rilonacept. Additionally, the majority (59%) of all patients who had intensified treatment to second-line corticosteroids subsequently transitioned to IL-1 pathway inhibition as third-line therapy, with the most common reason for transitioning therapy being sharp or piercing chest pain (72%), whereas transition from second-line IL-1 pathway inhibition to third-line corticosteroids was uncommon.

The use of IL-1 pathway inhibition earlier in the disease course represents a therapeutic advancement and a paradigm shift in the management of RP, as targeted immunomodulation enables greater clinical effectiveness with a more favorable safety profile than that associated with long-term corticosteroid-based or aspirin/NSAIDs/colchicine-based regimens.^8^, ^9^, ^10^^,^^14^^,^^15^^,^^37^, ^38^, ^39^ Earlier IL-1 pathway inhibition could decrease the impaired QoL associated with multiple recurrences and may decrease emergency care visits and hospitalizations.^40^ The algorithms presented in recent international expert position statements are in line with RESONANCE data; these data are emblematic of the impact that expert experience and real-world evidence can have in providing actionable data and guidance to cardiology practices more broadly, which may inform future therapeutic algorithms and advance the evidence-based management of RP.^16^^,^^17^

### Study limitations

All data were derived from an interval analysis of an unlocked database from an ongoing registry, and, as such, data may be missing, incomplete, and/or may change with future data cleaning, with the potential bias direction depending on whether errors are random (toward the null) or systematic (in either direction). Data for treatment intensification beyond third-line therapy were not available to report for this interval analysis due to ongoing data collection. Although the sample size is modest, this interval analysis represents the largest multicenter study in RP. Although external validity to community-based centers is limited because patients were enrolled from pericardial disease–dedicated programs, this approach is similar to other registries of rare cardiovascular diseases.^41^ Investigators were encouraged to enroll consecutive RP patients, but it was not protocol-mandated due to the detailed retrospective and prospective data collection necessary for these patients who had varying levels of support across sites. However, baseline patient characteristics within RESONANCE are similar to prior single-center observational studies and a prior multicenter study of RP patients treated with anakinra.^34^^,^^42^^,^^43^ Given that RESONANCE is the largest multicenter RP registry in both number of sites and patients enrolled, insights gathered may help to inform and improve decision-making for patients more broadly. Lastly, data related to clinical outcomes associated with this earlier-line use of IL-1 pathway inhibition are still being collected and analyzed, as are data related to patient-reported outcomes.

## Conclusions

RESONANCE, the largest multicenter, real-world registry of patients with RP at U.S.-based RP treatment centers, tracks the adoption of evidence-based treatment approaches through implementation science. In this first report of data from RESONANCE, RP was confirmed to have a multiyear duration, and almost half (44%) of all patients receiving aspirin/NSAIDs/colchicine required treatment escalation to second-line therapy. Prior to the FDA approval of rilonacept in RP, patients needing second-line therapy beyond aspirin/NSAIDs/colchicine had historically been treated with corticosteroids per prior guideline recommendations. In the years postapproval of rilonacept in RP, second-line management of patients failing aspirin/NSAIDs/colchicine has shifted increasingly toward IL-1 pathway inhibition rather than chronic corticosteroids. Most patients who initiated corticosteroids as second-line therapy subsequently transitioned to IL-1 pathway inhibition, whereas few patients treated with second-line IL-1 inhibitors received subsequent corticosteroids. Planned future analyses from RESONANCE related to long-term clinical events and patient-reported outcomes could further inform best practices and future therapeutic algorithms in the management of this debilitating disease.

Perspectives **COMPETENCY IN MEDICAL KNOWLEDGE:** Given the chronicity of recurrent pericarditis and the need for long duration of treatment, clinicians should be aware that prolonged corticosteroid therapy increases iatrogenic flares because the serial corticosteroid tapers necessary to mitigate the known metabolic side effects result in periodic subtherapeutic dosing and disease reactivation. Thus, clinicians should prioritize corticosteroid-sparing strategies, such as IL-1 pathway inhibition when treating recurrent pericarditis.

**TRANSLATIONAL OUTLOOK 1:** In patients with recurrent pericarditis failing aspirin/NSAIDs/colchicine, the evidence-based approach of second-line IL-1 pathway inhibition, instead of corticosteroids, could obviate the consequences of long-term corticosteroid use.

**TRANSLATIONAL OUTLOOK 2:** The use of IL-1 pathway inhibition as second-line therapy represents a therapeutic advancement, and a paradigm shift in the management of recurrent pericarditis, as targeted immunomodulation enables greater clinical effectiveness with a more favorable safety profile than that associated with long-term corticosteroid-based or aspirin/NSAIDs/colchicine-based regimens.

## Funding support and author disclosures

This study was funded by 10.13039/100016492Kiniksa Pharmaceuticals, Lexington, MA. Dr Cremer has received grants and consultant fees from 10.13039/100016492Kiniksa Pharmaceuticals; and has received grants and personal fees from Sobi. Dr Luis has received consultant fees from 10.13039/100016492Kiniksa Pharmaceuticals, Cardiol Therapeutics, and 10.13039/100004374Medtronic. Dr Garshick has received consultant fees from 10.13039/100002491BMS, Agepha, and 10.13039/100016492Kiniksa Pharmaceuticals. Dr Raisinghani has received consultant fees from 10.13039/100016492Kiniksa Pharmaceuticals. Dr Weber has received consultant fees from 10.13039/100016492Kiniksa Pharmaceuticals, Novo Nordisk, Horizon Therapeutics, and 10.13039/100002491BMS. Drs Clair, Parameswaran, Curtis, and Paolini are shareholders and employees of Kiniksa Pharmaceuticals. Dr Klein has received grants and consultant fees from 10.13039/100016492Kiniksa Pharmaceuticals, Cardiol Therapeutics, and 10.13039/100004319Pfizer. Ms Winnowski has reported that she has no relationships relevant to the contents of this paper to disclose.
