# Supplementary material for: IL-1 Pathway Inhibition in Recurrent Pericarditis Management: Real-World Adoption of Corticosteroid Sparing in RESONANCE
Source: JACC Adv. 2025 Aug 15;4(9):102050. doi: 10.1016/j.jacadv.2025.102050 (PMC12392773; doi:10.1016/j.jacadv.2025.102050)
Supplement: Supplemental Material [file mmc1.docx]

**SUPPLEMENTAL APPENDIX**

**Supplemental Methods**

In the inactive cohort, all patients eligible for inclusion had a physician-confirmed diagnosis of recurrent pericarditis, a recurrence occurring between 3-5 years prior to enrollment in RESONANCE, and confirmed resolution of recurrent pericarditis-related symptoms with no further recurrent pericarditis-treatment within the last 3 years prior to enrollment in RESONANCE. The target ratio of enrollment between the two cohorts was 90% for active patient cohort and 10% for inactive patient cohort, in order to capture a substantial amount of prospective and longitudinal data, while also enabling various comparisons between historical and contemporaneous datasets.

For inactive patients, the observation period was purely retrospective, occurring between 3-5 years prior to enrollment in RESONANCE, and captured only data pertaining to their last recurrent pericarditis episode (**Supplemental Figure 3**).

**Supplemental Figure 1. RESONANCE site locations**


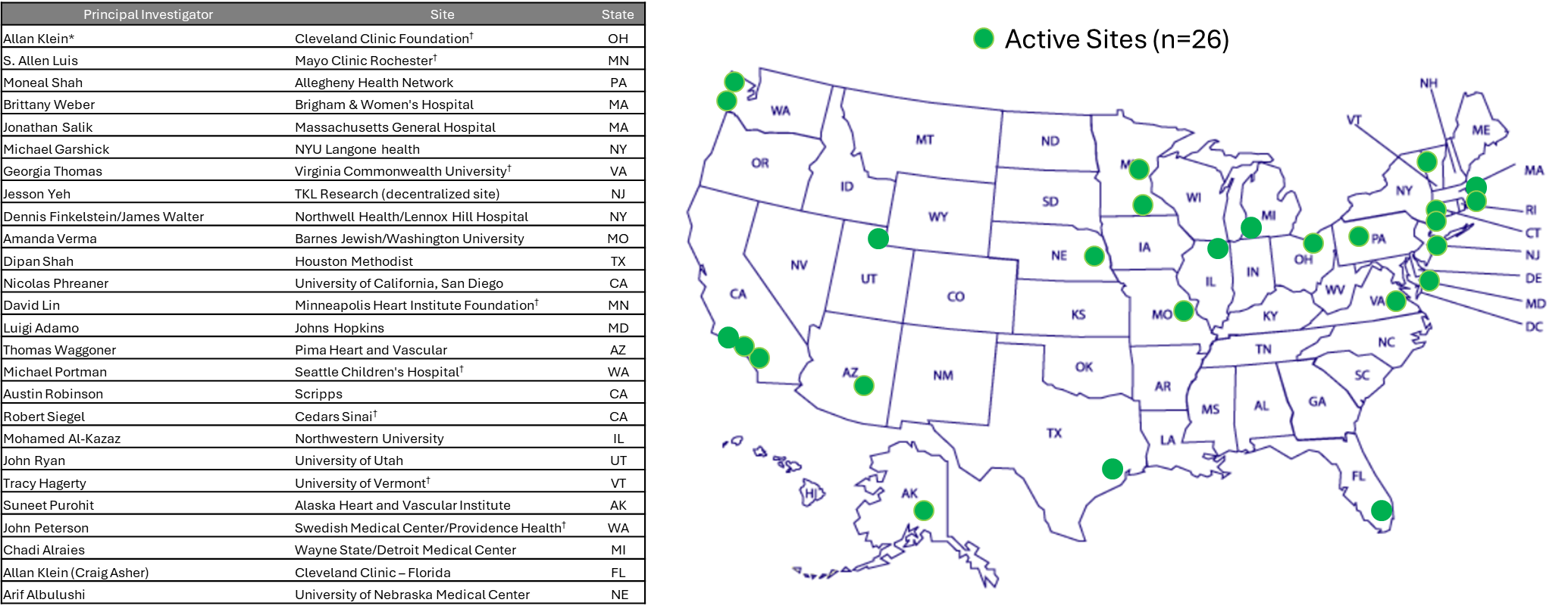
*North American RESONANCE Lead Coordinator

^†^Prior study site for RHAPSODY.

**Supplemental Figure 2. Example of visual patient narrative of real-world data collection
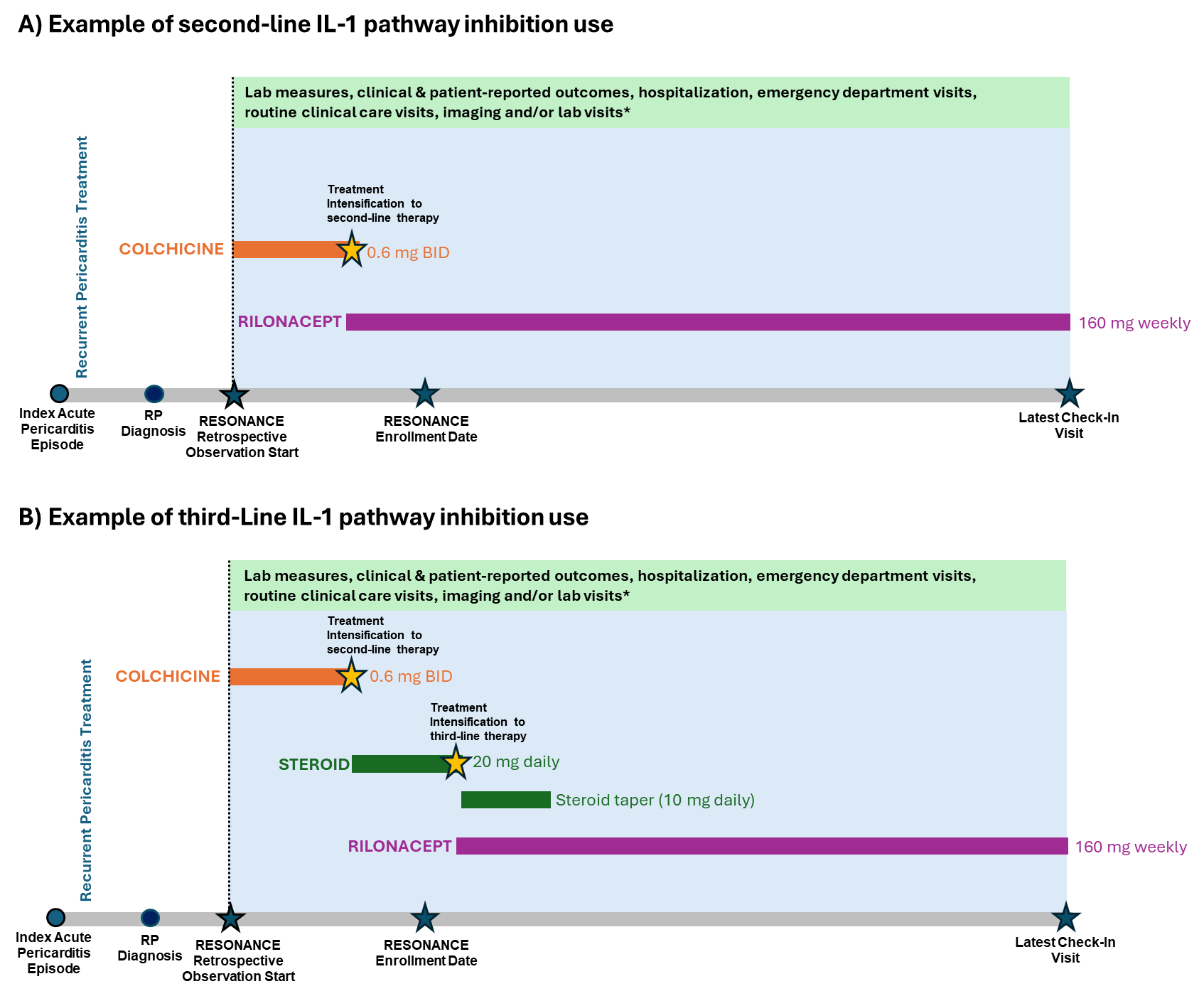
**

*Data currently being collected and not included in this interval analysis. Lab measures include C-reactive protein; imaging includes cardiac magnetic resonance imaging, echocardiogram, and computerized tomography scans; clinical & patient-reported outcomes include pericarditis recurrences, HCRU (hospitalization, ED visits, routine clinical care visits, imaging and/or lab visits), episode assessment questionnaire, PPNRS, PROMIS-29 +2 v2.1 or PROMIS Pediatric/Parent Proxy Profile 25, D-12, four items from the FSS, PGIPS, fear from episode items​​, and work-related disability.

BID, twice a day; D-12, Dyspnoea-12 questionnaire; FSS, Fatigue Severity Scale; HCRU, healthcare resource utilization; PGIPS; Patient Global Impression of Pericarditis Symptom Severity; PPNRS, pericarditis pain measured using numeric rating scale; PROMIS, patient-reported outcomes measurement information system; RP, recurrent pericarditis; SF-36; 36-Item Short Form Health Survey.**Supplemental Figure 3. Inactive cohort retrospective study design
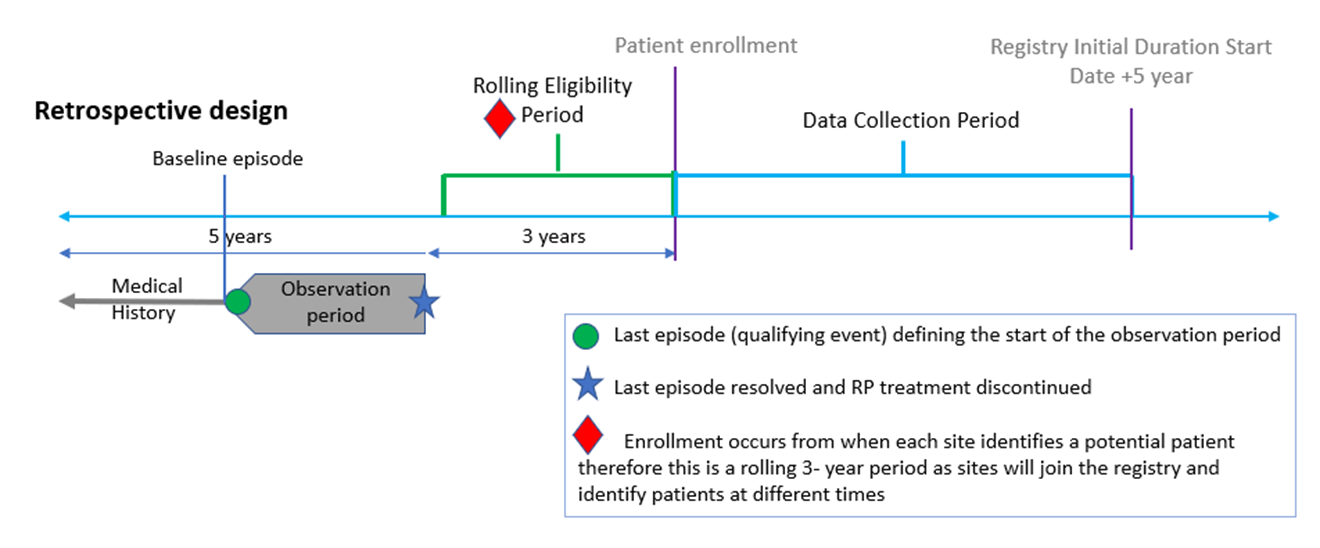
**

RP; recurrent pericarditis.

**Supplemental Table 1. Schedule of assessments for patients in the active cohort**

|  | **Medical History** | **Retrospective Observation Period**  **(12 months prior to registry enrollment or index acute pericarditis episode)** | **Baseline  (at enrollment)** | **Prospectively (every 6 months after enrollment)** |
| --- | --- | --- | --- | --- |
| Informed consent |  |  | **X** |  |
| Inclusion/Exclusion criteria |  |  | **X** |  |
| Patient characteristics (if change) |  |  | **X** | **X (if change)** |
| Sociodemographic information (if change) |  |  | **X** | **X (if change)** |
| Referral pattern to the site |  |  | **X** |  |
| Use or have access to a wearable device |  |  | **X** |  |
| Date of initial acute episode of pericarditis | **X** | **X** |  |  |
| Date of first recurrent pericarditis symptom(s) onset | **X** | **X** |  |  |
| Date of initial recurrent pericarditis diagnosis | **X** | **X** |  |  |
| Medication prescribed for the first recurrence, (including date and dose) | **X** |  |  |  |
| Primary cause of initial pericarditis episode, cause of post pericardial injury pericarditis | **X** |  |  |  |
| Total number of episodes, from first recurrence | **X** |  |  |  |
| Total number of episodes (start and end date if available) from start of retrospective observational period until baseline |  | **X** |  |  |
| Total number of episodes (start and end date if available) from last Soc visit or 6-month review |  |  |  | **X** |
| ICD code assigned for diagnosis of initial and recurrent pericarditis episode | **X** | **X** | **X** | **X** |
| Other relevant recurrent pericarditis medical and surgical history | **X** | **X** |  |  |
| Selected comorbidities | **X** | **X** | **X** | **X** |
| Medication(s) for relevant comorbidities | **X** | **X** | **X** | **X** |
| Clinical examination |  |  | **X** | **X** |
| Chief complaint at consultation |  |  | **X** | **X** |
| Primary cause of recurrent pericarditis episode, or symptoms worsening |  | **X** |  | **X** |
| Last episode date |  |  | **X** |  |
| PGA-PA |  |  | **X** | **X** |
| Findings of non-invasive diagnostic tests and cardiac monitoring |  | **X** | **X** | **X** |
| Findings from laboratory measures |  | **X** | **X** | **X** |
| Recurrent pericarditis medications for each episode: drug name, start and stop dates, reasons for initiation and cessation, doses, corticosteroids tapering |  | **X** | **X** | **X (if change or dose altered)** |
| Surgical pericardiectomy | **X** | **X** | **X** | **X** |
| Treating physician |  | **X** | **X** | **X** |
| Site characteristics |  |  | **X** |  |
| HCRU collected by the physician |  |  |  | **X** |
| Survival status |  |  |  | **X** |

HCRU, healthcare resource utilization; ICD, International Classification of Diseases; PGA-PA, Physician Global Assessment of Pericarditis Activity.

**Supplemental Table 2.** **Select patient and disease characteristics of patients intensifying treatment to second-line vs third-line IL-1 pathway inhibition**

|  | **Patients Intensifying to second-line IL-1 Pathway Inhibition**  **(n=76)** | **Patients Intensifying to third-line IL-1 Pathway Inhibition**  **(n=36)** | **P-value** |
| --- | --- | --- | --- |
| **Age*, years**; mean ± SD | 45.7 ± 15.6 | 44.6 ± 17.8 | 0.76 |
| **Female**, n (%) | 49 (64.5) | 14 (38.9) | 0.01 |
| **White**, n (%) | 64 (84.2) | 31 (86.1) | 0.79 |
| **Etiology**, n (%) |  |  | 0.87 |
| Idiopathic / viral pericarditis | 54 (71.1) | 26 (72.2) |  |
| Post-cardiac injury / post-procedural | 10 (13.2) | 4 (11.1) |  |
| Other causes | 1 (1.3)** | 2 (5.6)*** |  |
| Not reported / unknown / missing | 11 (14.5) | 4 (11.1) |  |
| **Pericarditis disease duration at RP diagnosis*,** years; median [Q1, Q3] | 0.2 [0.1, 0.4] | 0.2 [0.1, 0.4] | 0.42 |
| **Pericarditis disease duration at RESONANCE enrollment*,** years; median [Q1, Q3] | 1.7 [0.8, 4.0] | 1.0 [0.6, 2.4] | 0.98 |
| **Pericarditis disease duration at end of RESONANCE observation period***^†^**,** years; median [Q1, Q3] | 2.6 [1.6, 5.6] | 2.6 [1.8, 4.1] | 0.88 |
| **Observation period**, years, median [Q1, Q3]; sum | 2.0 [1.1, 2.8]; 147.7 | 2.2 [1.5, 2.9]; 75.8 | 0.38 |

*At index acute episode; disease duration calculated as time since index acute episode.

** Other causes include post COVID-19 booster (n=1).

*** Other causes include postoperative pericarditis (n=1) and post COVID vaccine (n=1).

^†^End of RESONANCE observation defined as end of study, last check-in visit, or December 31, 2023.

PY, patient-year; Q1, first quartile; Q3, third quartile; N/A, not applicable; SD, standard deviation.
